# Unlocking the potential of spring wheat genetic resources: uncovering resistance sources against leaf rust and yellow rust

**DOI:** 10.1007/s00122-026-05365-9

**Published:** 2026-09-06

**Authors:** Behnaz Soleimani, Anne-Kathrin Pfrieme, Ulrike Beukert, Heike Lehnert, Max Haupt, Axel Himmelbach, Martin Mascher, Nils Stein, Andreas Stahl, Frank Ordon, Jochen Christoph Reif, Albrecht Serfling

**Affiliations:** 1 Institute for Resistance Research and Stress Tolerance, Julius Kuehn-Institute (JKI)-Federal Research Centre for Cultivated Plants, Quedlinburg, Germany; 2 Present Address: Institute for Agricultural and Nutritional Sciences, Chair of Plant Breeding, Martin-Luther-University Halle-Wittenberg, Halle (Saale), Germany; 3 Institute for Biosafety in Plant Biotechnology, Julius Kuehn-Institute (JKI)-Federal Research Centre for Cultivated Plants, Quedlinburg, Germany; 4 Leibniz Institute of Plant Genetics and Crop Plant Research (IPK), Seeland, Germany; 5 Institute of Agricultural and Nutritional Sciences, Martin Luther University of Halle-Wittenberg, Halle (Saale), Germany

## Abstract

**Key message:**

Integrating high-throughput phenotyping with multiple complementary GWAS models reduces method-dependent bias and enables reliable identification of novel loci and elite germplasm for durable yellow and leaf rust resistance in wheat.

**Abstract:**

The causal agents of yellow and leaf rust in wheat, *Puccinia striiformis f.* sp.* tritici* and *Puccinia triticina*, pose a serious threat to grain yield and quality worldwide. Growing durable resistant wheat cultivars is an effective protection measure contributing to sustainable agriculture. Many of the known resistance genes, however, have been overcome due to the high genetic diversity and adaptability of pathogen populations. Therefore, the present study aimed to identify novel loci associated with yellow rust and leaf rust in a genome-wide association study using 1984 spring wheat accessions from the German Federal ex situ Genebank. Phenotypic data obtained from a detached-leaf assay were combined with 90,283 genotyping-by-sequencing genome-wide markers. Six significant peak marker-trait associations were identified for yellow rust and leaf rust, respectively. These are located on chromosomes 1D, 2B, 3B, 4A, 4B, 4D, 5A, 6B, and 7D. Six candidate genes were identified close to the identified loci. These findings may be valuable for identifying and deploying genetic resources to broaden the genetic basis of resistance and safeguard durability of resistance against yellow and leaf rust.

**Supplementary Information:**

The online version contains supplementary material available at https://doi.org/10.1007/s00122-026-05365-9.

## Introduction

Wheat (*Triticum aestivum* L.) is one of the world´s most important staple crops and provides food for approximately 35% of the global population. However, its yield and quality are severely threatened by fungal diseases such as leaf rust (LR), caused by *Puccinia triticina* and yellow rust (YR), caused by *P. striiformis* (Rampitsch et al. 2019). Both diseases can lead to substantial yield losses and negatively impact grain quality.

Due to the significant impact of rust diseases, breeding programs are focused on developing wheat cultivars with enhanced long-lasting resistance. Developing and cultivating disease-resistant wheat varieties is considered an effective, economical, and environmentally sustainable way to mitigate these losses. Resistance to fungal pathogens is generally classified as either race-specific (qualitative) resistance or non-race-specific (quantitative) resistance. To date, approximately 90 resistance genes/loci (Supplementary Table S7) have been identified for LR, and 83 (Supplementary Table S6) for YR (McIntosh et al. 1995; McIntosh et al. 2005; McIntosh et al. 2009; McIntosh et al. 2013; McIntosh et al. 2015; McIntosh et al. 2017; Baranwal 2022).

Most known resistance genes are race-specific, typically governed by single major genes, and confer effective protection during the seedling stage. This form of resistance is usually durable throughout the plant’s life and is often associated with a hypersensitive response (Bolton et al. 2008). While such resistance has proved valuable in breeding programs for European wheat varieties (Park et al. 2001; Pathan and Park 2006; Serfling et al. 2011), its durability is limited. Due to the high genetic diversity and evolutionary adaptability of *Puccinia* species (Dyck and Kerber 1985), new pathogen races can frequently overcome these resistance genes, rendering them ineffective over time. In contrast, non-race-specific resistance genes, often referred to as adult plant resistance, are generally controlled by multiple genes, providing broader and more durable protection.

Rust pathogens undergo rapid evolutionary changes, driven by high mutation/recombination rates, resulting in the recurrent emergence of races that can overcome deployed resistance genes. Pyramiding multiple resistance genes is reported as an effective strategy to enhance durable resistance against rust diseases, as combination of major resistance genes can reduce disease levels compared to single genes (Mundt 2018). Therefore, it is crucial to identify and combine resistance genes or loci, including those genes or loci that have not previously been used in breeding programs. To achieve the longest possible effectiveness of resistance, it is therefore essential to strategically incorporate previously unexploited resistance loci, thereby safeguarding and extending the durability of resistance. A key resource for achieving this goal is the genetic diversity preserved in genebanks. These collections contain a wide variety of plant genetic resources, including alleles and traits that have been lost or neglected in elite breeding lines. Many of these traits confer resistance to biotic stresses, such as rust and other significant diseases (Dinglasan et al. 2022).

Accurate phenotyping and genotyping are essential for the efficient use of genetic resources in resistance breeding. Traditional phenotyping methods, however, are expensive, labor-intensive, and often destructive, which limits the significance and accuracy of the results. Automated high-throughput phenotyping platforms, such as the Macrobot system (Lück et al. 2020a), have been developed to address these limitations. These platforms enable the rapid, standardized, and non-destructive analysis of disease resistance in large plant populations (Gill et al. 2022). Combining detached-leaf assays with robotics further enhances throughput and reliability (Beukert et al. 2021).

Technological progress in high-throughput genotyping complements advances in phenotyping by providing comprehensive insights into the plant genome and the genetic basis of complex agronomic traits. Molecular marker-assisted techniques have significantly accelerated resistance breeding. Examples of these strategies include marker-assisted recurrent selection (MARS; Johnson 2004; Eathington et al. 2007), marker-assisted gene pyramiding (Ragagnin et al. 2009; Costa et al. 2010), marker-assisted backcrossing (MABC; Hospital et al. 1992; Frisch et al. 1999; Septiningsih et al. 2009), and genomic selection (GS; Meuwissen et al. 2001; Bernardo 2010; Poland et al. 2012). However, modern plant breeding relies on high-throughput phenotyping and genotyping and the combination of these techniques provides comprehensive characterization of ex situ genebank resources (Volk et al. 2021), which allows identification of useful accessions in large and diverse ex situ collections which could be useful to develop cultivars with enhanced pathogen defense.

The development of high-throughput genotyping platforms including genotyping-by-sequencing (GBS) has made it possible to identify quantitative trait loci (QTL) and allelic variations underlying important traits through genome-wide association studies (GWAS) in various crops. Numerous studies in wheat have used GWAS to identify marker-trait associations (MTAs) conferring resistance to LR (El Messoadi et al. 2022; Kumar et al. 2020; Li et al. 2020a, b; Ahmed et al. 2021; Leonova et al. 2020; Vikas et al. 2022; Lhamo et al. 2023), and YR (Elbasyoni et al. 2017; Long et al. 2019; Yao et al. 2021; Vikram et al. 2021; Zhang et al. 2021; Qiao et al. 2024; Sharma et al. 2025). Recent large-scale genome-wide association studies (GWAS), have significantly expanded our understanding of the genetic basis of LR and YR resistance in wheat (Joukhadar et al. 2020; Lakkakula et al. 2025; Wu et al. 2025). Despite the advances in GWAS studies, continued examination of diverse germplasm collections is essential to confirm reported resistance loci, discover new sources of resistance, and more effectively exploit existing genetic diversity in breeding programs. In the present study, a diverse set of wheat genebank accessions covering a wide range of genetic diversity is evaluated. This approach, while enabling a comprehensive examination of resistance to leaf rust and yellow rust, helps to identify significant marker-trait associations (MTA) and sheds light on the genetic basis of resistance to these two economically important diseases.

The objectives of this study were (i) to evaluate the phenotypic variability for seedling resistance to *P. striiformis and P. triticina* in a large spring wheat genebank collection using a semi-automated, high-throughput phenotyping platform; (ii) to perform GWAS in order to identify genomic regions associated with resistance to these pathogens, (iii) to compare the detected associations with previously reported resistance loci; and (iv) to prioritize candidate genes within the identified QTL regions based on gene annotation, GO terms, transcriptome data, and sequence similarity to published resistance loci.

## Material and methods

### Plant material

The analyzed spring wheat collection comprised 1984 genetic resources (PGRs), which were provided as seed samples by the German ex situ genebank of the Leibniz Institute of Plant Genetics and Crop Plant Research (IPK, Gatersleben, Germany) and propagated according to the protocols outlined by Schulthess et al. (2021) and Hinterberger et al. (2022). A subset of 1,984 genotypes with complete LR and YR phenotypic and genotypic data was considered for GWAS and further analysis.

The genotypes originate from 65 countries (http://gbis.ipk-gatersleben.de/) including 215 genotypes from Africa, 207 from America (67, 68, and 72 from central, North, and South America, respectively), 1,040 from Asia, 432 from Europe, 28 from Australia and New Zealand, and 62 of unknown origin. This set represents a wide diversity in years of acquisition and growth habits (see Supplementary Table S1).

## High-throughput phenotyping of seedlings with assays for detached leaves

Due to the large number of genotypes evaluated, the detached-leaf assays were conducted in multiple greenhouse phenotyping batches using the semi-automated, high-throughput phenotyping platform Macrobot (Lück et al. 2020a, b). The experimental setup followed the detached-leaf assay workflow described by Beukert et al. (2021) and Hinterberger et al. (2023). Seeds were germinated and grown under greenhouse conditions for ten days until growth stage EC12. Up to seven primary leaf segments per genotype, each originating from an independent seedling, were collected and used as biological replicates. The number of replicates varied among genotypes due to differences in germination success and quality control of leaf segments. Leaf segments were transferred to agar plates and arranged according to the standard Macrobot workflow (Beukert et al. 2021; Hinterberger et al. 2023).

The leaf segments were inoculated with the aggressive leaf rust isolate 77WxR (Naz et al. 2008) and the aggressive yellow rust isolate WxYr27 (Rollar et al. 2021). The highly susceptible variety Borenos (registration year 1987, Strube Research, Söllingen, Germany) was used as a susceptible control line for LR trials, Akteur (registration year 2003, Deutsche Saatveredelung AG, Lippstadt, Germany) for YR trials. Automated and multimodal image analysis of the infected segments was performed using the Macrobot platform after an 8-day incubation for LR and a 15-day incubation for YR. The degree of infection was evaluated using the BlueVision software by quantifying the percentage of the infested leaf area as described by Lück et al. (2020a, b).

## Quality assessment of phenotypic data and estimation of degree statistics

A manual quality check was not feasible due to the large volume of data. Therefore, we implemented an automated, standardized data quality control pipeline as described by Hinterberger et al. (2022). All data processing and analysis were performed in the R statistical environment R (version 4.3.3; R Core Team 2024) following the methodology outlined by R Core Team (2024). According to Henderson (1975), a linear mixed model (Eq. 1) was fitted, and a nominal significance level of 0.05 was used to identify outliers based on model residuals.

1 $$y_{ijk} = \,\mu \, + \,g_{i} + \,e_{j} + \,t_{k} \left( {e_{j} } \right) + \,\smallint_{ijk}$$

In this model,* y* represents the percentage of infested leaf area, and* μ *denotes the global mean. *g*_*i*_ is the fixed effect of the *i*th genotype, e_*j*_ is the fixed effect of the *j*th experiment and t_*k*_ (e_*j*_) is the fixed effect of the *k*th tray, nested in the *j*th experiment and ϵ denotes the residual.

Residuals from this model were extracted for outlier detection. Data points with residuals exceeding the nominal significance threshold were classified as outliers and excluded from subsequent analyses. To estimate relationship between YR and LR, Pearson correlation coefficients were calculated between best linear unbiased estimates (BLUEs) after inoculation with LR and YR using R function cor(). To estimate the variance components of the phenotypic traits, model (1) was employed, specifying the overall mean *μ* as a fixed effect, while including all remaining terms as random effects. Genotype-specific BLUEs were derived using the same model, with genotype as a fixed effect (Nyquist and Baker 1991) and were subsequently used as phenotypic input for GWAS analyses. BLUES account for variation associated with experimental batches and tray effects while providing a single estimate of genotypic performance across replicated observations. All estimations of variance components and BLUEs were performed in the linear mixed model (Eq. 1) using ASReml-R software, version 4 (Butler et al. 2009).

Broad-sense heritability (H^2^) was calculated using the method described by Falconer and Mackay (1996), following equation:

2 $$H^{2} = \sigma^{2}_{{\mathrm{g}}} /\left( {\sigma^{2}_{{\mathrm{g}}} + \left( {\sigma^{2}_{{\mathrm{e}}} /R} \right)} \right)$$

where σ^2^_g_ represents the genetic variance among genotypes derived from Eq. 1, σ^2^_e_ represents the residual variance, and R represents the average number of replicates per genotype.

### Genotyping

Genotyping-By-Sequencing (GBS) was conducted as described previously (Zhang et al. 2024) using a two-enzyme (PstI and MspI) approach (Poland et al. 2012) and genomic DNA isolated from seedling tissue. In typical experiments, 288 individually barcoded samples were pooled and sequenced (Illumina NovaSeq 6000 device,122 cycles single read, one lane S1 flow cell, XP-workflow) at IPK-Gatersleben, yielding an average of 2.5 million reads per genotype. Prior to downstream analysis, adapter sequences and low-quality bases were removed from the raw sequence data prior to downstream analysis (Schulthess et al. 2021). Raw sequence processing was conducted as described previously for GBS read mapping and SNP calling (Schulthess et al. 2022) with the distinction that reads were aligned to the wheat genome assembly of Chinese Spring V2.1 (Zhu et al. 2021).

### Filtering of genome-wide marker data

The marker set was mapped to the Chinese Spring RefSeq V2.1 reference genome using physical positions. Markers that were not assigned to specific chromosomes (*n* = 2218) were removed. The remaining markers filtered to exclude those with ≥ 30% missing value. SNP imputation was performed using the Beagle software package (version 4.1; Browning, and Browning 2007, 2009). Subsequently, markers with a minor allele frequency (MAF) of ≤ 1% and heterozygosity ≥ 10% were removed from the imputed marker set. After these filtering steps, 90,283 GBS markers remained and were used to estimate population structure and kinship.

Population structure was analyzed using informative markers by Bayesian clustering within the STRUCTURE software (version 2.3.4 software; Pritchard et al. 2000). Ten independent runs were performed for each number of clusters (*K*) from one to ten. Each run consisted of 50,000 burn-in steps followed by 50,000 Markov Chain Monte Carlo (MCMC) iterations. The optimal number of subpopulations was determined using the Evanno ΔK method (Evanno et al. 2005), based on the rate of change in the log probability of the data between successive *K* values, as implemented in the STRUCTURE HARVESTER (version 2.3.4, Pritchard et al. 2000; Earl and vonHoldt 2012). Additionally, a principal coordinate analysis (PCoA) was performed using DARwin 6 software (Perrier and Jacquemoud-Collet 2006).

### Genome-wide association study (GWAS)

Phenotypic and genotypic data were used to perform GWAS and to identify marker-trait associations (MTAs) by using four different tools: TASSEL (Trait Analysis by Association, Evolution and Linkage, Bradbury et al. 2007), GAPIT (Genome Association and Prediction Integrated Tool, Lipka et al. 2012), FARMCPU (Fixed and Random Model Circulating Probability Unification, Liu et al. 2016) and GenABEL (Aulchenko et al. 2007) in R.

In both TASSEL and GAPIT, a compressed mixed linear model (CMLM) was used that incorporated a kinship (*K*) matrix and a population structure (*Q*) matrix as a correction factor for relatedness and population structure. FARMCPU used a fixed and random model circulating probability unification algorithm in R, and GenABEL used a mixed linear model containing both the kinship matrix (*K*) and the population structure matrix (*Q*) was used to identify significant MTAs. To illustrate the number of identified markers and also common markers between four different GWAS methods, a Venn diagram was created using R.

Initially, the Bonferroni–Holm-adjusted significance threshold of −log_10_ (*p* value) ≥ 6.3 at *α* = 0.05 was applied to identity significant MTAs. However, this stringent cutoff yielded only one significant marker for LR. To reduce the probability of detection of false-positive associations, we considered only MTAs which were detected by all four GWAS methods for downstream analysis. An additional, less stringent threshold (LOD ≥ 3) was therefore set to declare an MTA as significant. The identified significant markers were assigned to the QTL region based on linkage disequilibrium (LD) decay. QTL regions were defined as ± 2.6 Mb around each significant marker, corresponding to the LD decay (*r*^2^-based) estimated across all chromosomes. The LD decay was calculated as the squared allelic correlation (*r*^2^) between all pairs of markers within a chromosome using the “genetics” (Warnes et al. 2013) and “LDheatmap” (Shin et al. 2006) packages in R. The obtained *r*^2^ values were plotted against the estimated genetic distance between markers in base pairs. LD was determined by the intersection of the fitted locally weighted polynomial regression (LOESS) curve and critical *r*^2^ values. LD was calculated for each chromosome separately and across all chromosomes. Next, the identified markers were compared with those in previous studies to confirm and interpret the results of the present study. Later, identified MTAs were compared with previously reported QTLs to evaluate their overlap with reported genetic loci and identify potential new genomic regions. The novelty of QTL regions was assessed based on LD-defined genomic intervals (± 2.6 Mb) and comparison with previously reported QTLs and resistance loci.

Finally, candidate genes were identified by screening all flanking sequences of associated disease markers within ±2.6 Mb (corresponding to the LD decay) for published functional gene annotations of Chinese Spring (IWGSC RefSeq V2.1).

## Results

### *Response of wheat seedling to P. striiformis f.* sp.* tritici and P.triticina*

**Fig. 1:**
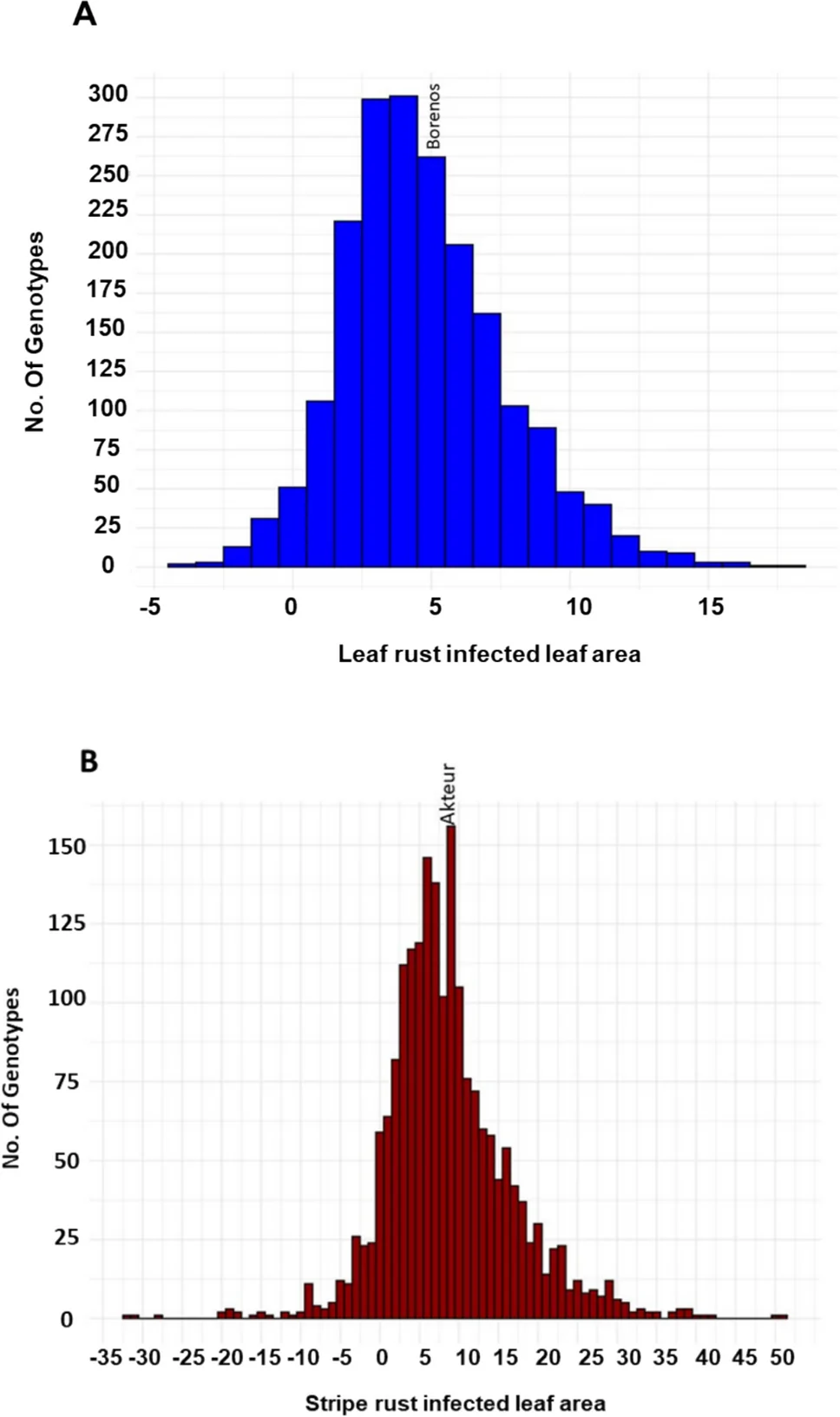
Distribution of the best linear unbiased estimates (BLUEs) of the percentage of infected leaf area of 1,984 spring wheat genetic resources (PGRs) after inoculation with leaf rust (**A)** and stripe rust (**B**)

A total of 1984 spring wheat genotypes were phenotyped using detached-leaf assays and the Macrobot system after being inoculated with yellow rust (YR) and leaf rust (LR) under greenhouse conditions. After quality control, for technical, experimental, and biological replicates BLUEs were calculated and their distributions showed a unimodal frequency pattern, which is consistent with a quantitative, polygenic inheritance of resistance. Raw phenotypic observations showed continuous, largely unimodal distributions for both traits (Supplementary Fig. 1), which were similar to the distributions observed for the BLUE values (Fig. 1A + B, Supplementary Tables S2 and S3).

Genotypes were considered as more resistant relative to the susceptible control variety if their BLUE value was below zero relative to the respective control (Borenos for LR, 5.4%; Akteur for YR, 9.9%) which was set as reference (0). A total of 1323 and 1287 genotypes exhibited lower infection rates for LR and YR, compared to the respective control variety. Among these, 875 genotypes exhibited lower infection rates than the controls for both rust diseases. To examine the relationship between LR and YR resistance, the Pearson correlation coefficient was calculated for the BLUE values, resulting in a significant, albeit weak, positive correlation (r = 0.14, *p* < 0.001).

**Fig. 2:**
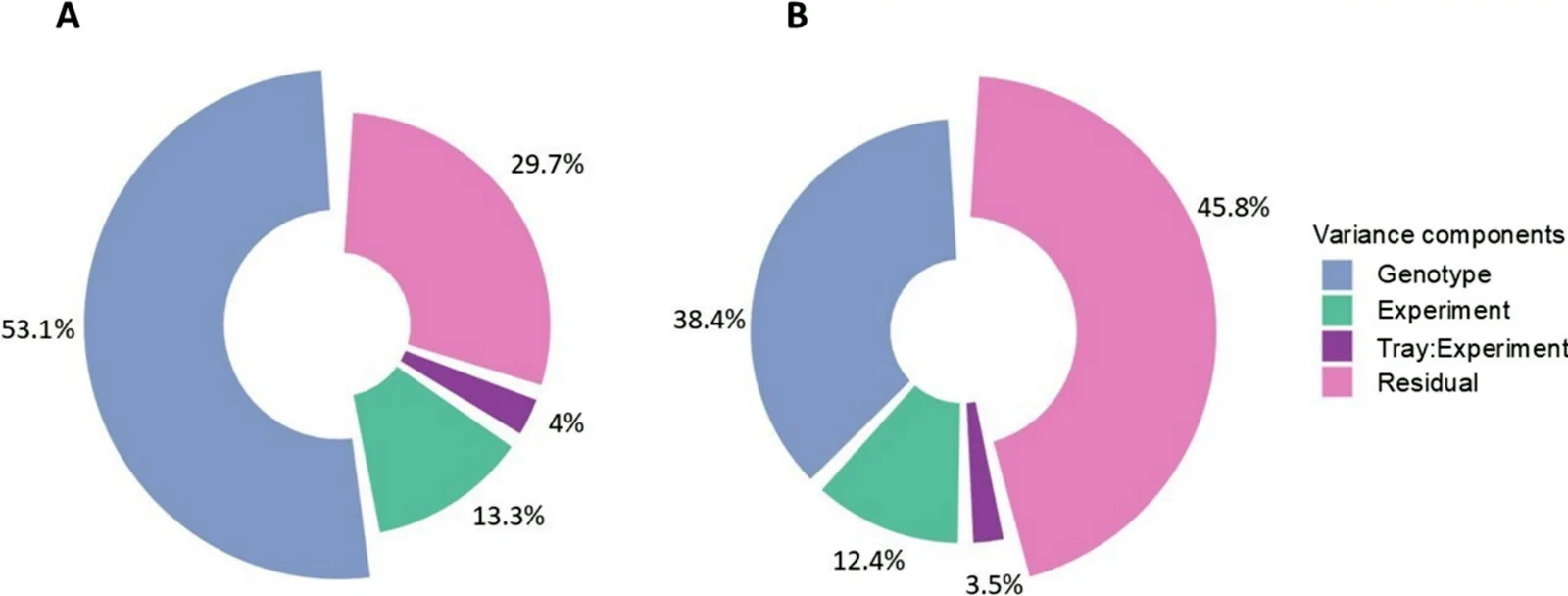
Variance component analysis of leaf rust (**A**) and stripe rust (**B**) infection. “Experiment” refers to the infection and screening batch, while “Tray” refers to all genotypes grown on a single tray in the greenhouse, nested within an infection and scanning batch. Error bars represent standard errors (SE) of the estimated variance components. All components (genotype, experiment, tray) were significant at α = 0.01

Analysis of variance components revealed a high genotypic variance and a broad-sense heritability H^2^= 0.54 for LR infection, indicating strong genetic determination of resistance. In contrast, high residual variance for YR infection led to a reduced heritability (H^2^ = 0.38), indicating increased unexplained variation compared to LR suggesting a higher influence of environmental factors or lower phenotyping accuracy in this case (Fig. 2).

### Genotyping, filtering and population structure

Genotyping 1984 wheat genotypes resulted in the identification of 1,021,664 GBS markers. After removing markers with more than 30% missing data and those without assigned chromosomal positions, 432,995 markers remained for further analysis. These markers were then filtered based on minor allele frequency (MAF ≤ 1%) and heterozygosity (≥ 10%), resulting in a dataset of 90,283 markers All of these markers were subsequently used for GWAS analyses without further selection. Only markers showing significant associations in the GWAS were selected for downstream analyses and interpretation. The number of markers per chromosome ranged from 965 (chromosome 4D) to 7,495 (chromosome 7 A) (Supplementary Fig. 2). The software Plink was used to identify 15,771 informative markers, which were then used to estimate population structure and kinship.

**Fig. 3:**
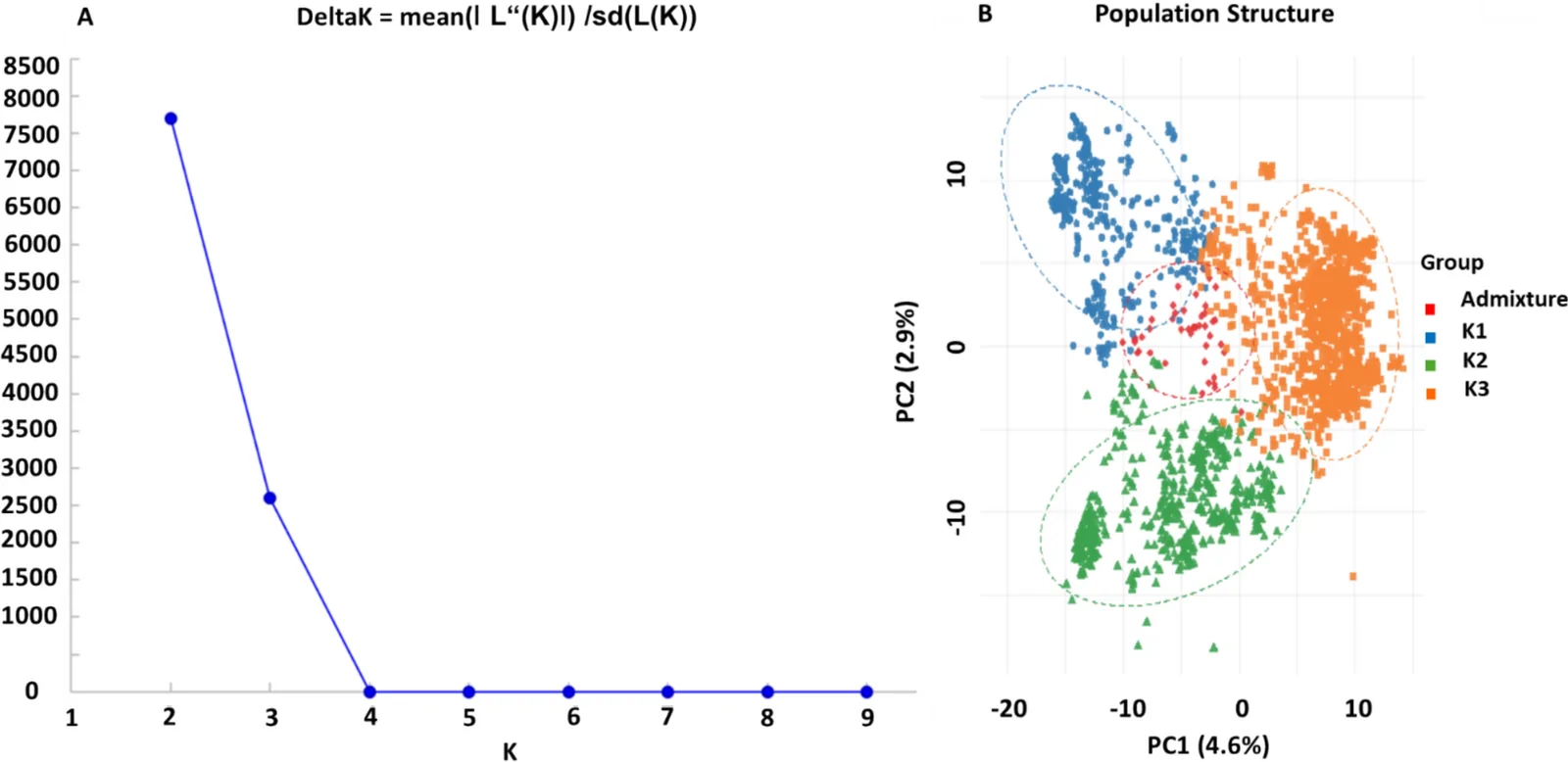
Population structure analysis of 1984 spring wheat based on 90,283 GBS markers, (**A**): Delta K plot obtained from Structure harvester. (**B**): Principal coordinates analysis (PcoA) plot indicated three clusters (K1, K2 and K3) based on Structure result

To estimate the population structure, a set of 15,771 informative markers was analyzed using the STRUCTURE software. Based on the ΔK method, the optimal number of three subpopulations (K=3) was determined (Fig. 3A). Genotypes were then assigned to clusters according to their membership coefficients; individuals with a coefficient of at least 0.5 for a particular cluster were assigned to the corresponding cluster (Fig. 3B). Of the 1984 genotypes, 1040 originated from Asia and these genotypes were distributed across all three identified clusters.

The first cluster (K1) comprised mainly Asian genotypes (379 genotypes, 97.7%), 67.3 of which were from Southern Asia. India contributed the largest number of genotypes to K1 (149 genotypes), followed by Eastern Asia, with 119 genotypes from China (31.4%) (Supplementary Fig. 3). Minor proportions originated from Africa (0.5%), America (0.5%), Australia (1%), and Europe (0.3%). The second subpopulation (K2) included 494 genotypes, 407 (82.4%) of which originated from Asia. Within this group, 75.6% were from Southern Asia, predominantly from Iran (Supplementary Fig. 3). Genotypes from Africa and Europe constituted 13.6% and 3.4% of the second cluster, respectively. The distribution of South Asian genotypes across both clusters (K1 and K2) might be due to variations in climatic conditions within the region. The third cluster (K3) contained 1,057 genotypes (53.3% of the total), which were more evenly distributed across the continents: Europe (38.6%), Asia (20.5%), America (19.3%), Africa (13.5%), and Australia (2.6%) (Supplementary Fig. 3).

Genotypes with a membership coefficient of less than 0.5 were classified as admixed (*n*=45), the majority of which were from Asia (37 genotypes, or 82.2%). Genotypes for which geographical origin was unknown made up 0.6% of K2 and 5.8% of K3 (Supplementary Fig. 3). The PCoA showed that the first and second principal coordinates explained 4.6% and 2.9% of the genetic variation, respectively.

### Genome-wide association study (GWAS)

To identify marker-trait associations for LR and YR, different GWAS models were performed. The most suitable model was selected based on QQ plots (Supplementary Fig. 4) and calculation of mean squared difference (MSD, Supplementary Table S4). The CMLM (*Q*+*K*) model provided the most appropriate control of population structure and relatedness and selected for YR. For LR, CMLM (K) model was selected, because inclusion of population structure covariates did not substantially improve model performance.

The identified peak markers were assigned to QTL regions based on their physical chromosomal position, which was estimated using the LD decay (as ±2.6 Mb). The LD varied between 946,330.7 bp (chromosome 6B) and 8,950,806 (chromosome 2D). A LD of 2,614,573 was calculated across all 21 chromosomes. The QTL region was adjusted to ±2.6 million base pairs from each identified significant marker based on LD decay across all chromosomes. MTAs, with LOD≥ 3 (− log10 (*p* value)) considered significant and identified MTAs by all four methods, were considered reliable (Table 1).

**Table 1:** Summary of significant marker-trait associations for stripe rust (YR) and leaf rust (LR) using four GWAS methods (LOD (− log10(*p* value), minor allele frequency (MAF ≤ 1%) and effect of FARMCPU is presented in the Table)

Trait	Marker	Chrom	Pos	*P*.value	LOD	MAF	Effect
YR	Chr1D_8677328	1D	8,677,328	8.34E−08	7.08	0.3	0.93
YR	Chr2B_660133069	2B	660,133,069	2.51E−04	3.6	0.02	2.05
**YR**	**Chr4A_110976029**	**4A**	**110976029**	**1.01E-08**	**8**	**0.32**	**0.98**
YR	Chr6B_51649618	6B	51649618	9.68E−04	3.01	0.18	0.7
YR	Chr6B_54841657	6B	54841657	7.58E−04	3.12	0.18	0.72
**YR**	**Chr6B_243663920**	**6B**	**243663920**	**5.41E−16**	**15.27**	**0.03**	**−3.74**
**LR**	**Chr3B_53685308**	3B	**53685308**	**1.74E−07**	**6.76**	**0.03**	**1.21**
**LR**	**Chr4B_554311160**	4B	**554311160**	**4.83E−05**	**4.32**	**0.02**	**−0.99**
**LR**	**Chr4D_15502361**	4D	**15502361**	**1.06E−15**	**14.98**	**0.01**	**−1.89**
**LR**	**Chr4D_396886837**	4D	**396886837**	**3.58E−05**	**4.45**	**0.01**	**−0.82**
**LR**	**Chr5A_459494322**	5A	**459494322**	**1.25E−04**	**3.9**	**0.07**	**−0.42**
**LR**	**Chr7D_611401245**	7D	**611401245**	**8.31E−26**	**25.08**	**0.01**	**3.24**

Markers identified for the first time for YR and LR are shown in bold format

**Fig. 4:**
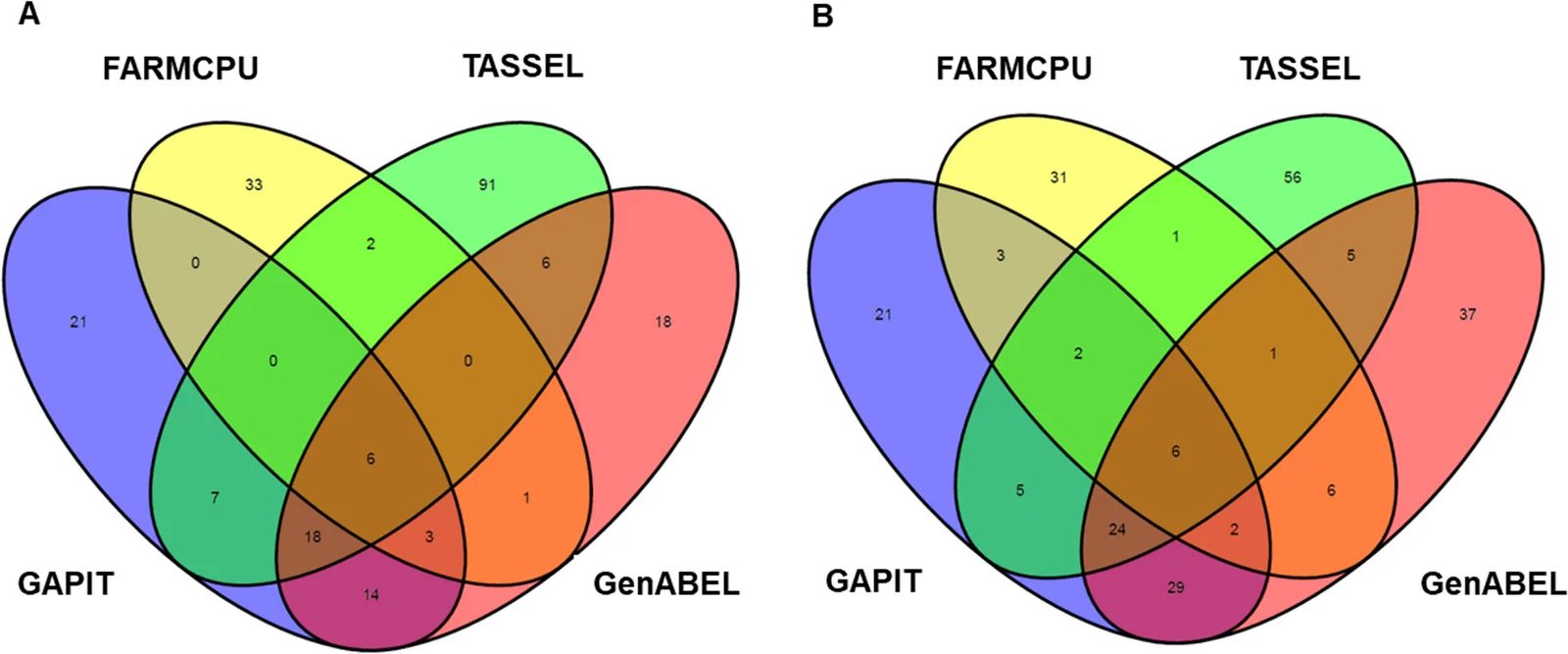
Venn diagrams indicating the overlapping of significant peak marker-trait associations for leaf rust (**A**) and stripe rust (**B**) using four different statistical methods for GWAS

**Fig. 5:**
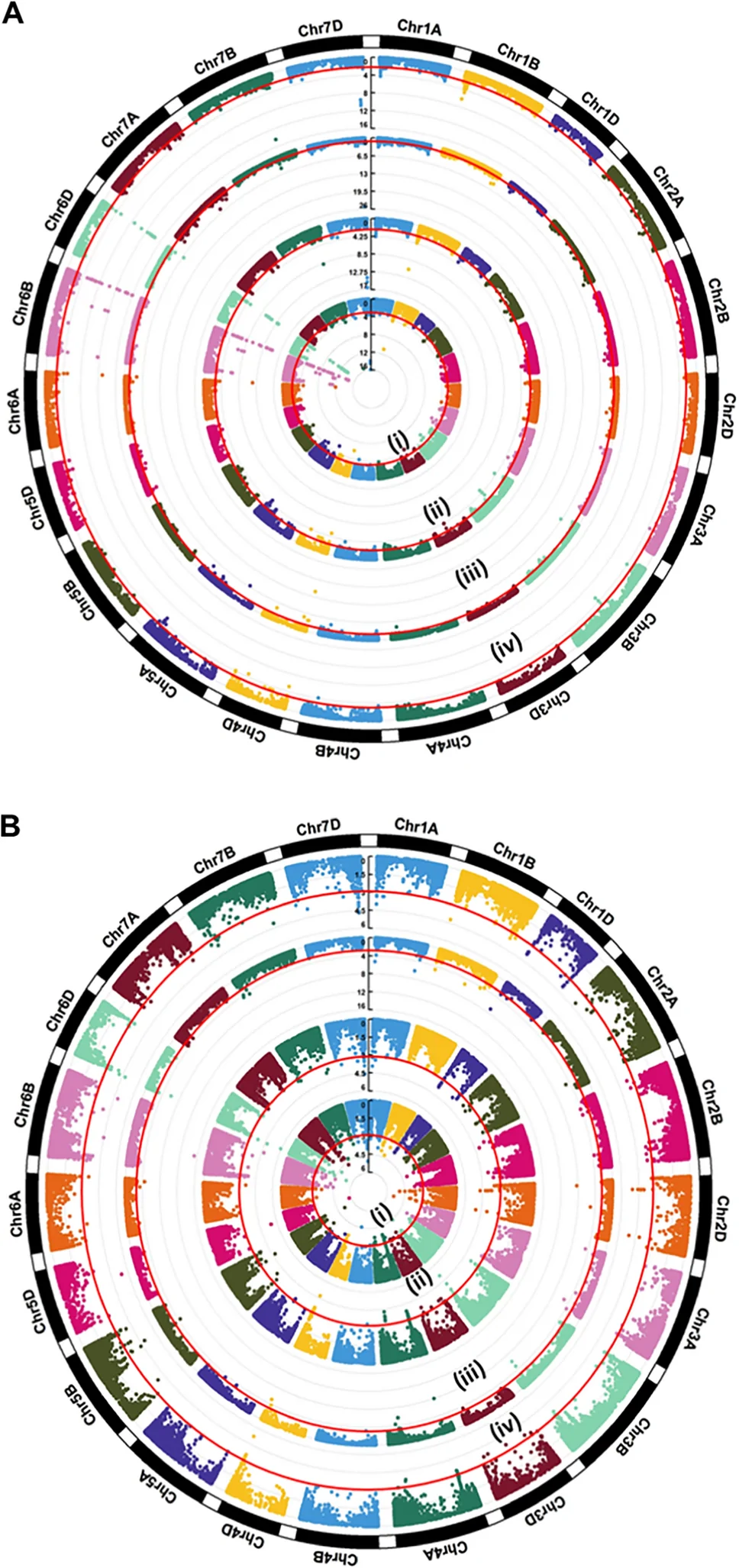
Circular Manhattan displaying results obtained by four different GWAS methods: (i) CMLM (in TASSEL), (ii) CMLM (in GAPIT), (iii) FarmCPU, and (iv) GenABEL for leaf rust (LR, **A**), stripe rust (**B**). The red line (LOD = 3, (− log10(*p* value)) indicates threshold for identifying significant marker-trait associations

For LR, 130, 69, 45, and 66 peak markers were identified using TASSEL, GAPIT, FARMCPU and Gen ABEL, respectively, across all wheat chromosomes (Fig. 4A and 5A as well as supplementary Table S5). GAPIT and Gen ABEL showed the highest number of common LR peak markers (41) across all wheat chromosomes, except for chromosomes 1D, 2B, 2D, 3D, 5D, and 6D.

FARMCPU had relatively few common markers with the other methods used. For example, 10 common peak markers were shared between FARMCPU and GenABEL, 9 were shared between FARMCPU and GAPIT, and 31 markers were shared between GAPIT and TASSEL. All four methods identified six markers for LR (Fig. 4A) on chromosomes 3B, 4B, 4D, 5 A, and 7D, with two markers on chromosomes 4D (Table 1).

For YR, 100, 92, 52, and 110 peak markers were identified using TASSEL, GAPIT, FARMCPU, and GenABEL, respectively (Figs. 4B and 5B, as well as Supplementary Table S5). GenABEL detected the highest number of peak markers for YR, which were distributed across all wheat chromosomes except 4B. GenABEL and TASSEL shared 36 peak markers across nearly all chromosomes, except 1 A, 3 A, 4B, 5 A, and 7A. A total of 61 common peak markers were identified between GAPIT and GenABEL on 19 chromosomes. GenABEL and FARMCPU shared 15 common peak markers on chromosomes 1 A, 1D, 2 A, 2B, 3B, 4 A, 4D, 5B, 6B, 7 A, and 7B. TASSEL and GAPIT shared 37 markers distributed on chromosomes 1D, 2 A, 2B, 2D, 3B, 3D,4A, 4D, 5B, 5D, 6B, 6D, 7 A, 7B, and 7D. Ten markers were identified between TASSEL and FARMCPU on seven wheat chromosomes (1D, 2B, 2D, 4 A, 5B, 6B, and 6D). Thirteen markers were identified as common markers between GAPIT and FARMCPU on chromosomes 1D, 2B, 2D, 4 A, 4B, 5 A, 6B, 6D, 7B.

In total, six peak markers were found to be common to all four methods used (Fig. 4B). These markers were located on six wheat chromosomes (1D, 2B, 4 A, 5B, and 6B), with chromosome 6B comprising three identified markers for YR (Table 1).

These results indicate that although each GWAS method identifies unique associations, a subset of peak markers is consistently identified by different methods and therefore, provides reliable candidate loci for further analysis. Furthermore, chromosomes 6B (for YR) and 4D (for LR) have multiple stable peaks, indicating these regions as potential biological importance.

The significant peak markers identified in this study were compared with previously reported QTLs and genomic regions associated with resistance to YR and LR (Supplementary Tables S6 and S7, respectively). In the present study, GBS markers were aligned to reference genome Chinese Spring v2.1, while previous studies relied on different genotyping platforms (array-based SNP platforms) and different versions of the reference genome. Therefore, direct comparisons were performed with studies aligned to the Chinese Spring v2.1 reference genome, whereas studies based on different reference genome versions were compared using overlapping chromosomal regions or QTL intervals. of identical SNP markers was not possible, and comparisons were performed based on overlapping chromosomal regions or QTL intervals. For YR, six MTAs were also identified, of which four “Chr1D 8677328,” “Chr2B_660133069,” “Chr6B_51649618,” and “Chr6B_54841657” were located within genomic regions on chromosomes 1D, 2B and 6B that co-localize with previously reported YR resistance QTLs (Zhu et al. 2023; Liu et al. 2018; Jia et al. 2020; Vazquez et al. 2015; Huang et al. 2021; Cheng et al. 2022; El Messoadi et al. 2022; Zhou et al. 2022; Luo et al. 2005; and Wu et al. 2025).

For LR, six MTAs were located in regions of the genome that had not previously been reported to be associated with LR resistance and were described here for the first time. To investigate the possible functional relevance of the associated markers, the flanking sequences of all LR and YR associated markers were mapped to the reference genome sequence to identify nearby high-confidence (HC) genes. For YR, a total of 5952, 5204, 3547, and 6820 HC genes were located in the vicinity of significant markers detected by TASSEL, GAPIT, FARMCPU and Gen ABEL, respectively (Supplementary Table S8). For LR, 6867, 3697, 2670, 3690 HC genes were identified for associated markers by TASSEL, GAPIT, FARMCPU and GenABEL, respectively (Supplementary Table S9). Six candidate HC genes were found for YR and six for LR among the markers that were commonly identified by all four methods (Table 2). Some of these genes have functional annotations related to plant defense and stress responses. (Fig. 5).

**Table 2:** List of identified high-confidence genes for identified common marker-trait associations (MTAs) for stripe rust (YR) and leaf rust (LR)

Trait	Chr^a^	Position	Marker	gene ID	Description
YR	1D	8,677,328	Chr1D_8677328	TraesCS1D03G0036900	UDP-glycosyltransferase
YR	2B	660,133,069	Chr2B_660133069	TraesCS2B03G1160100	p-loop containing nucleoside triphosphate hydrolases superfamily protein, putative
**YR**	**4A**	**110976029**	**Chr4A_110976029**	**TraesCS4A03G0204200**	**Cysteine-rich receptor-kinase-like protein**
YR	6B	51649618	Chr6B_51649618	TraesCS6B03G0166000	NBS-LRR-like resistance protein
YR	6B	54841657	Chr6B_54841657	TraesCS6B03G0172900	Ubiquitin thioesterase
**YR**	**6B**	**243663920**	**Chr6B_243663920**	**TraesCS6B03G0510000**	**Serine/threonine-protein phosphatase 2 A regulatory subunit B'' subunit alpha**
**LR**	**3B**	**53685308**	**Chr3B_53685308**	**TraesCS3B03G0165900**	**Mitochondrial glycoprotein**
**LR**	**4B**	**554311160**	**Chr4B_554311160**	**TraesCS4B03G0732600**	**Heat shock protein**
**LR**	**4D**	**15502361**	**Chr4D_15502361**	**TraesCS4D03G0054700**	**Lysine–tRNA ligase**
**LR**	**4D**	**396886837**	**Chr4D_396886837**	**TraesCS4D03G0569000**	**CAAX amino terminal protease family protein**
**LR**	**5A**	**459494322**	**Chr5A_459494322**	**TraesCS5A03G0614500**	**Basic helix-loop-helix transcription factor**
**LR**	**7D**	**611401245**	**Chr7D_611401245**	**TraesCS7D03G1191900**	**receptor kinase 1**

The bold highlighted markers, indicating novel identified MTAs for YR and LR in the present study

^a^^:^ chromosome

### Identification of reliable resistance-associated loci

**Fig. 6:**
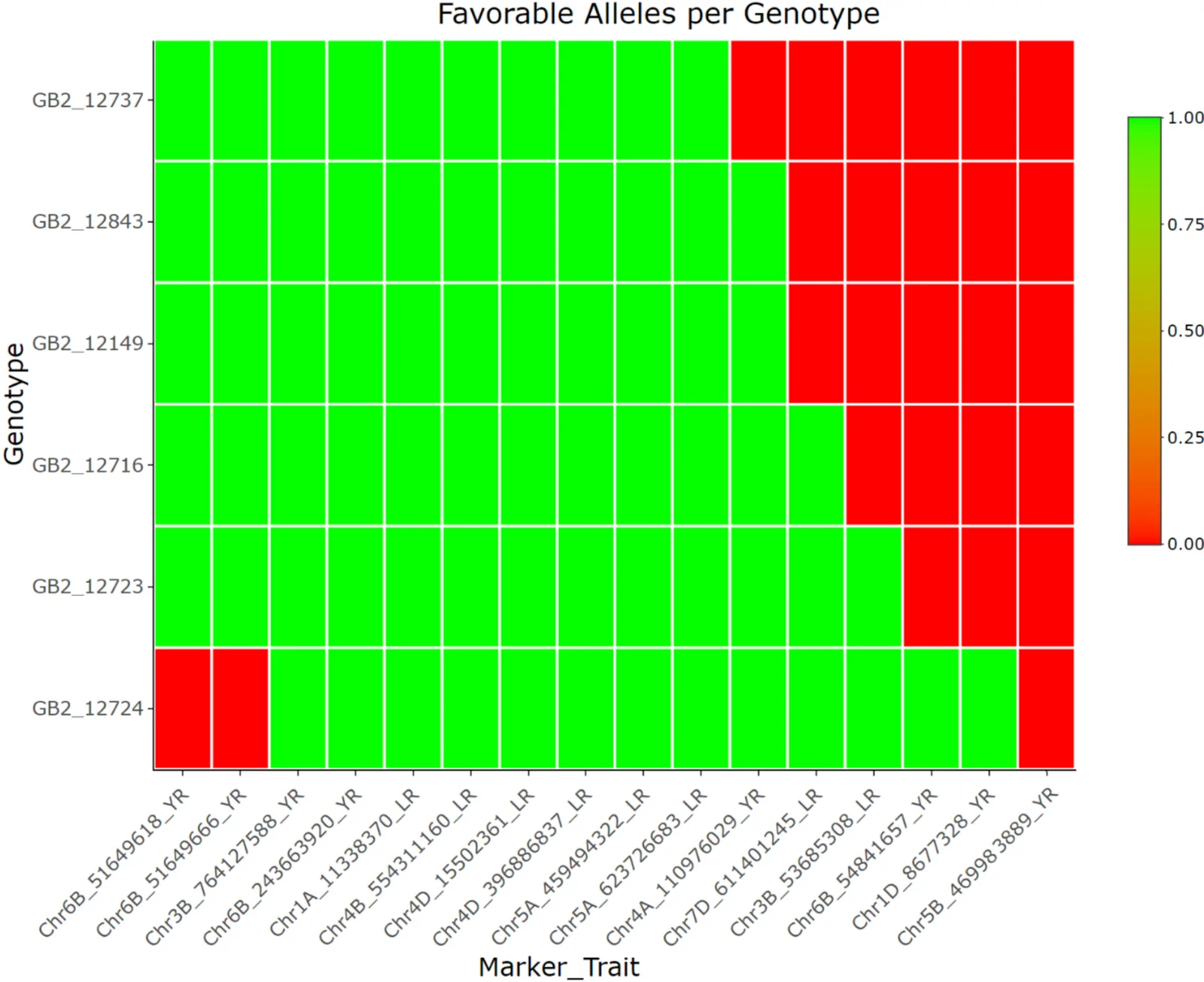
Incidence matrix of allele effects of identified markers for leaf rust (LR) and stripe rust (YR) in six genotypes (carrying favorable alleles for both LR and YR). Green (1): Favorable allele present (negative effect allele associated with reduced disease severity) and Red (0): Unfavorable allele present (positive effect allele associated with increased disease severity)

According to the resistance test of 1,984 genotypes, a set of 113 and 50 genotypes showed lower infection (BLUE values < 0) for YR and LR, respectively, compared to control genotypes. Among these sets, six genotypes were observed carrying favorable alleles for both LR and YR, originating from three distinct geographic regions (Europe (3), Asia (2) and America (1)). Therefore, favorable resistance alleles exist in the germplasm of different continents which point out the potential of combining resistance from diverse genetic resources to increase rust resistance. To uncover the genetic factors underlying YR and LR resistance, BLUEs were linked with allele effects from GWAS. The favorable alleles (allele with negative effect value from GWAS output) which reduced disease severity may be considered as promising genetic resources to improve the durability of rust resistance in breeding programs. Incidence matrix according to allele-effect indicates six genotypes have multiple favorable alleles for both rusts (Fig. 6).

## Discussion

In the present study, several novel MTAs for LR and YR were identified in a diverse panel of 1984 spring wheat genotypes. Of these, two MTAs for YR and six MTAs for LR were not previously reported and could provide novel targets for wheat breeding programs. Candidate genes associated with these MTAs are involved in plant defense pathways and represent potential mechanisms of resistance to rust diseases. Notably, some genotypes carried a combination of several unknown QTLs for LR and YR, which may confer potential resistance and persistence to evolving pathogen populations. These findings indicate the effectiveness of combining high-throughput phenotyping with sequencing-based genotyping (GBS) and GWAS in elucidating the genetic architecture of disease resistance in wheat. Considering that resistance conferred by major, race-specific genes can be rapidly overcome by new pathogenic strains, identified MTAs in the present study may serve as valuable resources for developing wheat cultivars with more stable and long-term resistance. Therefore, these results highlight novel genomic regions associated with rust resistance and provide practical strategies for using diverse germplasm to enhance durable resistance in wheat breeding programs.

### Limitations and future perspectives

One limitation of this study is that disease phenotyping was performed at the seedling stage under controlled greenhouse conditions, using single, specific invasive isolates for leaf rust (77WxR) and yellow rust (WxYr27). Disease severity was measured as the percentage of leaf area infected using a high-throughput imaging system (Macrobot). Although this approach provides accurate and quantitative data suitable for genome-wide association analyses, it is unable to distinguish classical patterns of infection and may not fully reflect the true diversity of the pathogen under field conditions. Therefore, complementary evaluations with multiple pathogen isolates and field trials are necessary to confirm the stability and extent of resistance of the identified loci.

### Comparative perspectives on rust resistance in spring and winter wheats

Spring and winter wheats can differ significantly in their genetic architecture of rust resistance due to differences in ecological adaptation, breeding history, and selection pressures. Winter wheat populations have typically evolved under different environmental conditions and pathogen pressures than spring wheats, which can lead to distinct patterns in the distribution of resistance genes and QTLs. Despite significant overlap at many resistance loci between these two groups, previous studies have reported differences in the abundance and efficiency of some rust resistance genes between spring and winter wheat germplasm (Kolmer 2013; Singh et al. 2011). Accordingly, the present study, which focused on a diverse panel of spring wheats, represents only a fraction of the genetic diversity associated with resistance in the wheat gene pool. Furthermore, genomic studies have shown the existence of population structure and distinct genetic differentiation between spring and winter wheat breeding populations, supporting somewhat distinct patterns in the distribution of resistance alleles (Reif et al. 2005; Walkowiak et al. 2020). Therefore, comprehensive comparative studies involving both growth types are necessary to more accurately identify common and specific resistance loci in the wheat gene pool.

### Distinct genetic bases for leaf rust and yellow rust resistance

The present study investigated a diverse set of 1984 spring wheat genotypes regarding their resistance to LR and YR. The unimodal distribution of infection rates observed after inoculation with YR and LR is consistent with quantitative variation in resistance. Similar observations have been reported in populations with polygenic resistance (Kolmer 2013; Poland et al. 2012), although continuous phenotypic distributions alone do not prove the underlying genetic architecture. Although seedling resistance can in some cases be controlled by major race-specific genes resulting in discrete phenotypic classes, the present dataset showed continuous phenotypic distributions and multiple loci of small to moderate effect. Furthermore, the raw phenotypic observations showed continuous distributions similar to those obtained from the BLUE values (Supplementary Tables S2 and S3), indicating that BLUE estimation did not substantially alter the overall distribution of the trait. Consequently, BLUE estimation is unlikely to have masked major resistance and was considered appropriate for the GWAS analyses performed in this study.

It is important to distinguish between seedling-stage resistance and adult-plant resistance when interpreting the identified loci. The present study evaluated resistance exclusively at the seedling stage using single isolates of *P. triticin*a and *P. striiformis* under controlled conditions. Therefore, the detected loci should primarily be interpreted as loci associated with seedling resistance. While seedling resistance is often associated with race-specific major genes, quantitative variation can also occur at this developmental stage (Ellis et al. 2014; Kolmer 2013). Conversely, adult-plant resistance is often quantitative and may provide broader and more durable protection across pathogen populations. Because no adult-plant evaluations or multi-isolate validation experiments were conducted, the present data do not allow conclusions regarding the developmental-stage specificity or race specificity of the identified loci.

The heritability estimated for LR (H^2^ = 0.54) was higher than that observed for YR (H^2^ = 0.38) and is consistent with previous studies using the same phenotyping platform (Beukert et al. 2021). This indicates a stronger genetic influence relative to residual variance for LR than for YR, although a substantial proportion of the phenotypic variation remained unexplained. The lower heritability observed for YR was primarily driven by increased residual variance. Because phenotyping was performed under controlled greenhouse conditions using a standardized detached-leaf assay and automated image analysis, large environmental fluctuations are unlikely to fully explain this pattern. Instead, the elevated residual variance may reflect a combination of biological and technical factors. First, YR assessments required a substantially longer incubation period (15 days) than LR assessments (8 days), which may have increased variation associated with detached-leaf stability and tissue senescence. Second, differences in inoculation efficiency among leaf segments may have contributed additional variation despite the standardized inoculation procedure. Third, differences in symptom development and colony morphology may influence the robustness of automated image-based disease quantification. Finally, isolate-specific biological characteristics of the YR isolate may have contributed to variation in infection success and disease development.

Although the increased residual variance reduced the heritability of YR resistance, it is more likely to reduce the statistical power to detect true marker-trait associations than to generate false-positive associations. To minimize the risk of spurious results, only marker-trait associations consistently identified by all four GWAS approaches were considered for further interpretation. Nevertheless, additional resistance loci with smaller effects may have remained undetected due to the lower heritability of the trait.

A significant, albeit weak, positive correlation (r = 0.14) was found between BLUEs for LR and YR, suggesting that resistance to these two diseases is largely governed by different genetic factors. This is in accordance with previous studies, which have shown that cross-resistance between LR and YR can occur but is generally limited in wheat germplasm. While some colocalized or pleiotropic QTL conferring resistance to multiple rusts have been reported (Ponce-Molina et al. 2018; Ye et al. 2022; Tong et al. 2024), the low correlation observed here suggests that these loci are not predominant in this panel.

### Methodological advances and model performance in GWAS

We performed GWAS to investigate the genetic basis of resistance genes to YR and LR. Here, a single-locus model such as compressed Mixed Linear Model (CMLM)) and multilocus models (FARMCPU and Gen ABEL) were applied to identify markers associated with YR and LR resistance. Multilocus models can overcome limitations of single-locus models for complex traits which are controlled by multiple loci simultaneously and reduce false-positive associations. As reported by Kaler et al. (2020), multilocus models are suitable for detecting traits which are controlled by many small effect loci. All approaches provided suitable model fits based on QQ-plots and MSD values. However, GAPIT demonstrated slightly improved performance for both traits.

### Comparison of markers with previous studies

In the present study, four out of 6 identified QTL regions for YR, namely QTL1_YR, QTL2_YR, QTL4_YR and QTL5_YR are overlapping with previously reported QTL regions (Supplementary Table S6). In particular, three studies have reported markers located in close proximity to Chr1D_8677328, at physical distances of 580,935 bp (Zhu et al. 2023), 251,642 bp (Zhang et al. 2021), and 86,080 bp (Liu et al. 2018), respectively. Similarly, two markers on chromosome 2B were previously identified by Luo et al. (2005) and Zhou et al. (2022) located at a distance of 601,820 bp and 974,478 bp from the identified MTA Chr2B_660133069 in the present study, respectively. In addition, two MTAs on chromosome 6B (Chr6B_51649618 and Chr6B_54841657) were identified in close proximity to a previously reported QTL by Wu et al. (2025), at distances of approximately 1.3 Mb and 1.9 Mb, respectively. QTL regions common between the present and previous studies confirm our GWAS results and refer to stable and durable resistance loci which can be useful for marker-assisted breeding. In contrast, no previously reported QTL were found adjacent to the MTAs identified for LR, suggesting that these loci likely represent novel genomic regions associated with leaf rust resistance.

### Functional insights into candidate genes underlying rust resistance

Markers that were consistently detected by all four GWAS models were prioritized for candidate gene analysis. Candidate genes were selected based on their physical proximity to significant markers within the LD-defined QTL intervals and their functional annotation in the reference genome. These genes were analyzed based on available genome annotations to investigate their known or potential roles in plant stress responses and defense processes. In total, six high-confidence candidate genes associated with yellow rust (YR) and leaf rust (LR) resistance were identified. As this study is based on association mapping, all functional interpretations are descriptive and do not imply causality.

The first gene of interest, potentially involved in YR resistance was associated with Cysteine-rich receptor-kinase-like protein (CRK). CRKs belong to subfamily of receptor-like kinases which play important roles in pathogen recognition and the activation of defense signaling pathways. For instance, *TaCRK10* as a sensor of *P. striiformis f.* sp.* tritici* and resistance to yellow rust through regulating nuclear processes (Wang et al. 2021). In addition, CRKs have been reported to positively regulate resistance to *P. triticina* in wheat through regulating the hypersensitive response and defense gene expression (Gu et al. 2020; Wang et al. 2021). Previous studies reported the distribution of CRKs on all wheat chromosomes except 4 A, 4B, and 4D (Liu et al. 2024). In contrast, the present study identified a CRK-encoding gene on chromosome 4A. This discrepancy might be explained by annotation or genotype-specific genomic variation.

NBS-LRR proteins in plants, by participating in complex signaling networks, induce a wide range of defense responses, including oxidative burst, changes in ion and calcium flux, activation of mitogen-activated protein kinase (MAPK) cascades, induction of genes related to disease response, and generation of hypersensitive responses (McHale et al. 2006). In the present study, NBS_LRR was identified on chromosome 6B at physical position 51,661,310 bp, which was associated with YR resistance.

Basic helix-loop-helix (bHLH) transcription factors constitute a large family of plant transcriptional regulators characterized by a conserved basic DNA binding region and a helix-loop-helix domain that enables protein dimerization and specific recognition of DNA motifs such as the E-box (CANNTG) (Song et al. 2025). These transcription factors are reported to be involved in the control of numerous processes, including growth and development, hormone signaling, metabolism, and biotic stress responses. Plant responses to biotic stresses include regulation of defense-related gene expression, interference with hormonal signaling pathways including jasmonic acid and salicylic acid, and modulation of immune responses resulting from pathogen recognition. For instance, response to LR includes changes in expression of transcription factor families such as WRKY, NAC, bZIP and potentially bHLH (Wang et al. 2019). The bHLH was identified on chromosome 5 A (at physical position 459,494,243 bp) which was associated to LR.

Receptor kinase 1 belongs to the family of receptor-like kinases (RLKs), which play a key role in pathogen recognition and activation of plant immune responses. RLKs function as pattern recognition receptors (PRRs) and recognize pathogen-associated molecular patterns (PAMPs). This recognition leads to the initiation of pattern-mediated immunity (PTI) and ultimately involves the production of reactive oxygen species (ROS), activation of MAPK pathways, and transcriptional reprogramming of defense genes (Boller and Felix 2009). In wheat, leaf rust resistance gene Lr10 encodes a receptor-like kinase (LRK10) is co-segregated with rust resistance, indicating the role of receptor-dependent signaling defense against *P. triticina* (Feuillet et al. 2003). Therefore, receptor kinase 1 identified in the present study might contribute to LR resistance through early pathogen recognition and activation of defense signaling pathways.

The QTL regions identified in this study span approximately 2.6 Mb, reflecting local linkage disequilibrium patterns. This resolution allows the identification of positional candidate genes based on their physical proximity to significant peak markers but does not permit definitive assignment of causality to individual genes. Further fine-mapping, allelic diversity analyses, and functional validation studies will be required to confirm the role of these candidate genes in yellow rust and leaf rust resistance.

### Limitations and implications for wheat improvement

Genetic validation is essential to confirm the involvement of candidate genes in wheat’s resistance to rust diseases before performing functional validation. Limitations of this study include the potential environmental effects on phenotyping accuracy and the complex genetic backgrounds of the wheat panel, which may have influenced the detection of associations. Therefore, future research should focus on conducting additional genetic validations of these loci, followed by their functional characterization and integration them into marker-assisted selection to improve disease resistance in wheat.

## Conclusion

In this study, the effectiveness of combining high-throughput phenotyping with genotyping data and employing multiple GWAS models to elucidate the genetic architecture of resistance to YR and LR in a diverse set of spring wheat was demonstrated. Analysis of the 1984 genetic resource using four complementary GWAS methods led to the identification of stable and reliable marker-trait associations for both diseases, including several novel and previously unreported loci. The simultaneous use of multiple GWAS approaches highlights the efficacy of this analytical framework for specifically investigating complex disease resistance traits, by reducing method-dependent bias and increasing confidence in the identified MTAs. Furthermore, the identification of genotypes with the accumulation of several unknown YR and LR resistance QTLs demonstrates the potential of this set of genotypes to achieve stable resistance. These results not only expand our understanding of the genetic basis of rust resistance in wheat, but also clearly demonstrate the practical utility of the high-throughput GWAS platform in identifying novel resistance loci and elite germplasm that can be used in wheat breeding programs.

## Supplementary Information

Below is the link to the electronic supplementary material.

Supplementary file1 (XLSX 2073 KB)

Supplementary file2 (XLSX 2575 KB)

Supplementary file3 (XLSX 7979 KB)

Supplementary file4 (XLSX 8804 KB)

## Data Availability

The phenotypic datasets generated for this study are available in the Supplementary Materials. Genotyping-by-sequencing (GBS) reads are available at the European Nucleotide Archive (ENA) under study PRJEB93924.”

## References

[CR1] Ahmed HGMD, Iqbal MN, Iqbal MA, Zeng Y, Ullah A, Iqbal M, Ikram RM (2021) Genome wide association mapping through 90K SNP array against leaf rust pathogen in bread wheat genotypes under field conditions. J King Saud Univ Sci 33:101628

[CR2] Aulchenko YS, Ripke S, Isaacs A, van Duijn CM (2007) GenABEL: an R library for genome-wide association analysis. Bioinformatics 23:1294–1296 17384015 10.1093/bioinformatics/btm108

[CR4] Baranwal D (2022) Genetic and genomic approaches for breeding rust resistance in wheat. Euphytica 218:159. 10.1007/s10681-022-03111-Y

[CR5] Bernardo R (2010) Genomewide selection with minimal crossing in self-pollinated crops. Crop Sci 50:624–627

[CR6] Beukert U, Pfeiffer N, Ebmeyer E, Hinterberger V, Lück S, Serfling A, Ordon F, Schulthess AW, Reif JC (2021) Efficiency of a seedling phenotyping strategy to support European wheat breeding focusing on leaf rust resistance. Biology (Basel) 10:628. 10.3390/biology10070628 34356483 PMC8301088

[CR7] Boller T, Felix G (2009) A renaissance of elicitors: perception of microbe-associated molecular patterns and danger signals by pattern-recognition receptors. Annu Rev Plant Biol 60:379–406 19400727 10.1146/annurev.arplant.57.032905.105346

[CR8] Bolton MD, Kolmer JA, Garvin DF (2008) Wheat leaf rust caused by *Puccinia triticina*. Mol Plant Pathol 9:563–575 19018988 10.1111/j.1364-3703.2008.00487.x PMC6640346

[CR9] Bradbury PJ, Zhang Z, Kroon DE, Casstevens TM, Ramdoss Y, Buckler ES (2007) TASSEL software for association mapping of complex traits in diverse samples. Bioinformatics 23:2633–2635 17586829 10.1093/bioinformatics/btm308

[CR10] Browning SR, Browning BL (2007) Rapid and accurate haplotype phasing and missing-data inference for whole-genome association studies by use of localized haplotype clustering. Am J Hum Genet 81:1084–1097 17924348 10.1086/521987 PMC2265661

[CR11] Browning BL, Browning SR (2009) A unified approach to genotype imputation and haplotype-phase inference for large data sets of trios and unrelated individuals. Am J Hum Genet 84:210–223 19200528 10.1016/j.ajhg.2009.01.005 PMC2668004

[CR12] Butler DG, Cullis BR, Gilmour AR, Gogel BJ, Thompson R (2009) ASReml-R reference manual. Version 4 VSN International Ltd. Hemel Hempstead, UK

[CR98] Cheng P, Zheng T, Li G, Wang M, Chen X, Kang Z (2022) Identification of high-temperature resistance to stripe rust and molecular detection of Yr genes in Chinese core collections of common wheat. Crop Prot 155:105934

[CR13] Costa MR, Tanure JPM, Arruda KMA, Carneiro JES, Moreira MA, Barros EG (2010) Development and characterization of common black bean lines resistant to anthracnose, rust and angular leaf spot in Brazil. Euphytica 176:149–156

[CR14] Dinglasan E, Periyannan S, Hickey LT (2022) Harnessing adult-plant resistance genes to deploy durable disease resistance in crops. Essays Biochem 66:571–580 35912968 10.1042/EBC20210096 PMC9528086

[CR15] Dyck PL, Kerber ER (1985) Resistance of the race-specific type. In: Diseases, distribution, epidemiology, and control, pp 469–500. Academic Press

[CR16] Earl DA, vonHoldt BM (2012) Structure harvester: a website and program for visualizing structure output and implementing the Evanno method. Conserv Genet Resour 4:359–361. 10.1007/s12686-011-9548-7

[CR17] Eathington SR, Crosbie TM, Edwards MD, Reiter RS, Bull JK (2007) Molecular markers in a commercial breeding program. Crop Sci 47(S3):S154–S163

[CR18] El Messoadi K, El Hanafi S, Gataa ZE, Kehel Z, Bouhouch Y, Tadesse W (2022) Genome wide association study for stripe rust resistance in spring bread wheat (*Triticum aestivum* L.). J Plant Pathol 104:1049–1059

[CR19] Elbasyoni I, El-Orabey WM, Baenziger PS, Eskridge K (2017) Association mapping for leaf and stem rust resistance using worldwide spring wheat collection. Asian J Biol 4:1–25

[CR20] Ellis JG, Lagudah ES, Spielmeyer W, Dodds PN (2014) The past, present and future of breeding rust resistant wheat. Front Plant Sci 5:641. 10.3389/fpls.2014.00641 25505474 PMC4241819

[CR21] Evanno G, Regnaut S, Goudet J (2005) Detecting the number of clusters of individuals using the software structure: a simulation study. Mol Ecol 14:2611–2620. 10.1111/j.1365-294X.2005.02553.x 15969739

[CR22] Falconer DS, Mackay TFC (1996) Introduction to quantitative genetics 4th edition Longman Harlow, UK

[CR23] Feuillet C, Kerlan MC, Mohr M, Leroy P, Choulet F, Boudet N, Sourdille P, Bernard M, Bernard S, Dedryver F, Dossat C, Rigaill G, Charmet G, Chalhoub B, Keller B (2003) Map-based isolation of the leaf rust disease resistance gene Lr10 from bread wheat. Science 278:833–836

[CR24] Frisch M, Bohn M, Melchinger AE (1999) Comparison of selection strategies for marker-assisted backcrossing of a gene. Crop Sci 39:1295–1301

[CR25] Gill T, Gill SK, Saini DK, Chopra Y, de Koff JP, Sandhu KS (2022) A comprehensive review of high throughput phenotyping and machine learning for plant stress phenotyping. Phenomics 2:156–183 36939773 10.1007/s43657-022-00048-z PMC9590503

[CR26] Gu J, Sun J, Liu N, Sun X, Liu C, Wu L, Liu G et al (2020) A novel cysteine-rich receptor-like kinase gene, TaCRK2, contributes to leaf rust resistance in wheat. Mol Plant Pathol 21:732–746 32196909 10.1111/mpp.12929 PMC7170779

[CR27] Henderson CR (1975) Best linear unbiased estimation and prediction under a selection model. Biometrics 31:423–447. 10.2307/2529430 1174616

[CR28] Hinterberger V, Douchkov D, Lück S, Kale S, Mascher M, Stein N, Reif JC, Schulthess AW (2022) Mining for new sources of resistance to powdery mildew in genetic resources of winter wheat: detached leaf phenotyping and association mapping. Front Plant Sci 13:836723. 10.3389/fpls.2022.836723 35300015 PMC8922026

[CR29] Hinterberger V, Douchkov D, Lueck S, Reif JC, Schulthess AW (2023) High-throughput imaging of powdery mildew resistance of the winter wheat collection hosted at the German Federal ex situ Genebank for Agricultural and Horticultural Crops. Gigascience 12:giad007 10.1093/gigascience/giad007 PMC9984986 36869695

[CR30] Hospital F, Chevalet C, Mulsant P (1992) Using markers in gene introgression breeding programs. Genetics 132:1199–1210 1459437 10.1093/genetics/132.4.1199 PMC1205240

[CR31] Huang S, Liu S, Zhang Y, Xie Y, Wang X, Jiao H, Wu S, Zeng Q, Wang Q, Singh RP, Bhavani S, Kang Z, Wang C, Han D, Wu J (2021) Genome-wide wheat 55K SNP-based mapping of stripe rust resistance loci in wheat cultivar Shaannong 33 and their alleles frequencies in current Chinese wheat cultivars and breeding lines. Plant Dis 105:1048–1056. 10.1094/PDIS-07-20-1516-RE 32965178

[CR32] Jia M, Yang L, Zhang W, Rosewarne G, Li J, Yang E, Chen L, Wang W, Liu Y, Tong H, He W, Zhang Y, Zhu Z, Gao C (2020) Genome-wide association analysis of stripe rust resistance in modern Chinese wheat. BMC Plant Biol 20:491. 10.1186/s12870-020-02693-w 33109074 PMC7590722

[CR33] Johnson R (2004) Marker-assisted selection. Plant Breed Rev 24:293–309

[CR34] Joukhadar R, Hollaway G, Shi F et al (2020) Genome-wide association reveals a complex architecture for rust resistance in 2300 worldwide bread wheat accessions screened under various Australian conditions. Theor Appl Genet 133:2695–2712. 10.1007/s00122-020-03626-9 32504212

[CR35] Kaler AS, Gillman JD, Beissinger T, Purcell LC (2020) Comparing different statistical models and multiple testing corrections for association mapping in soybean and maize. Front Plant Sci 10:1794 32158452 10.3389/fpls.2019.01794 PMC7052329

[CR36] Kolmer JA (2013) Leaf rust of wheat: pathogen biology, variation and host resistance. Forests 4:70–84

[CR37] Kumar D, Kumar A, Chhokar V, Gangwar OP, Bhardwaj SC, Sivasamy M, Prasad SS, Prakasha TL, Khan H, Singh R, Sharma P (2020) Genome-wide association studies in diverse spring wheat panel for stripe, stem, and leaf rust resistance. Front Plant Sci 11:748 32582265 10.3389/fpls.2020.00748 PMC7286347

[CR38] Lakkakula IP, Kolmer JA, Sharma R et al (2025) Identification of leaf rust resistance loci in hard winter wheat using genome-wide association mapping. Plant Genome 18:e20546. 10.1002/tpg2.20546 39757138 PMC11700927

[CR39] Leonova IN, Skolotneva ES, Salina EA (2020) Genome-wide association study of leaf rust resistance in Russian spring wheat varieties. BMC Plant Biol 20(Suppl 1):135. 10.1186/s12870-020-02333-3 33050873 PMC7557001

[CR40] Lhamo D, Sun Q, Zhang Q, Li X, Fiedler JD, Xia G, Faris JD, Gu YQ, Gill U, Cai X, Acevedo M, Xu SS (2023) Genome-wide association analyses of leaf rust resistance in cultivated emmer wheat. Theor Appl Genet 136:20. 10.1007/s00122-023-04281-6 36683081

[CR41] Li H, Wei C, Meng Y, Fan R, Zhao W, Wang X, Yu X, Laroche A, Kang Z, Liu D (2020a) Identification and expression analysis of some wheat F-box subfamilies during plant development and infection by *Puccinia triticina*. Plant Physiol Biochem 155:535–548 32836199 10.1016/j.plaphy.2020.06.040

[CR42] Li J, Jiang Y, Yao F, Long L, Wang Y, Wu Y, Li H, Wang J, Jiang Q, Kang H, Li W (2020b) Genome-wide association study reveals the genetic architecture of stripe rust resistance at the adult plant stage in Chinese endemic wheat. Front Plant Sci 11:625 32582233 10.3389/fpls.2020.00625 PMC7289992

[CR43] Lipka AE, Tian F, Wang Q, Peiffer J, Li M, Bradbury PJ, Gore MA, Buckler ES, Zhang Z (2012) Genome association and prediction integrated tool. Bioinformatics 28:2397–2399 22796960 10.1093/bioinformatics/bts444

[CR44] Liu X, Huang M, Fan B, Buckler ES, Zhang Z (2016) Iterative usage of fixed and random effect models for powerful and efficient genome-wide association studies. PLoS Genet 12:e1005767 26828793 10.1371/journal.pgen.1005767 PMC4734661

[CR45] Liu W, Naruoka Y, Miller K, Garland-Campbell KA, Carter AH (2018) Characterizing and validating stripe rust resistance loci in US Pacific Northwest winter wheat accessions (*Triticum aestivum* L.) by genome-wide association and linkage mapping. Plant Genome 11:170087 29505636 10.3835/plantgenome2017.10.0087 PMC12809922

[CR46] Liu H, Li X, Yin Z, Hu J, Xie L, Wu H, Han S et al (2024) Identification and characterization of the CRK gene family in the wheat genome and analysis of their expression profile in response to high temperature-induced male sterility. PeerJ 12:e17370 38737737 10.7717/peerj.17370 PMC11086307

[CR47] Long L, Yao F, Yu C, Ye X, Cheng Y, Wang Y, Wu Y, Li J, Wang J, Jiang Q, Li W (2019) Genome-wide association study for adult-plant resistance to stripe rust in Chinese wheat landraces (Triticum aestivum L) from the Yellow and Huai River Valleys. Front Plant Sci 10:596 31156668 10.3389/fpls.2019.00596 PMC6532019

[CR48] Lück S, Strickert M, Lorbeer M, Melchert F, Backhaus A, Kilias D, Seiffert U, Douchkov D (2020a) Macrobot”: an automated segmentation‑based system for powdery mildew disease quantification. Plant Phenomics. 10.34133/2020/5839856 33313559 PMC7706317

[CR49] Lück S, Beukert U, Douchkov D (2020b) BluVision Macro a software for automated powdery mildew and rust disease quantification on detached leaves. J Open Source Softw 5:2259. 10.21105/joss.02259

[CR97] Luo PG, Ren ZL, Zhang HQ, Zhang HY (2005) Identification, chromosomal location, and diagnostic markers for a new gene (YrCN19) for resistance to wheat stripe rust. Phytopathol 95(11):1266–1270 10.1094/PHYTO-95-1266 18943356

[CR50] McHale L, Tan X, Koehl P, Michelmore RW (2006) Plant NBS-LRR proteins: adaptable guards. Genome Biol 7:212 16677430 10.1186/gb-2006-7-4-212 PMC1557992

[CR51] RA McIntosh C Wellings RF Park 1995 Wheat rusts: an atlas of resistance genes CSIRO Publishing Melbourne

[CR52] McIntosh RA, Devos KM, Dubcovsky J, Rogers WJ, Morris CF, Appers R, Anderson OS (2005) “Catalogue of gene symbols for wheat: 2005 supplement.” Annual Wheat Newsletter 55:256–278

[CR53] McIntosh RA, Dubcovsky J, Rogers WJ, Morris CF, Xia XC (2009) Catalogue of gene symbols for wheat 2009 Supplement. https://shigen.nig.ac.jp/wheat/komugi/genes/macgene/supplement2009.pdf. Accessed 6 Mar 2020

[CR54] McIntosh RA, Dubcovsky J, Rogers WJ, Morris CF, Appels R, Xia XC (2013) Wheat gene catalogue 2013. https://wheatpw.usda.gov/GG2/Triticum/wgc/2013/. Accessed 16 Jan 2019

[CR55] McIntosh RA, Dubcovsky J, Rogers WJ, Morris CF, Appels R, Xia XC (2015) Catalogue of gene symbols for wheat 2015–2016 Supplement https://shigen.nig.ac.jp/wheat/komugi/genes/macgene/supplement2015.pdf. Accessed 6 Mar 2020

[CR56] McIntosh RA, Dubcovsky J, Rogers WJ, Morris CF, Appels R, Xia XC (2017) Catalogue of gene symbols for wheat 2017 Supplement. https://shigen.nig.ac.jp/wheat/komugi/genes/macgene/supplement2017.pdf. Accessed 6 Mar 2020

[CR57] Meuwissen THE, Hayes BJ, Goddard ME (2001) Prediction of total genetic value using genome-wide dense marker maps. Genetics 157:1819–1829 11290733 10.1093/genetics/157.4.1819 PMC1461589

[CR96] Mundt CC (2018) Pyramiding for resistance durability: Theory and practice. Phytopathol 108(7):792–802 10.1094/PHYTO-12-17-0426-RVW 29648947

[CR58] Naz AA, Kunert A, Lind V, Pillen K, Léon J (2008) AB-QTL analysis in winter wheat: II Genetic analysis of seedling and field resistance against leaf rust in a wheat advanced backcross population. Theor Appl Genet 116:1095–1104 18338154 10.1007/s00122-008-0738-y PMC2358941

[CR59] Nyquist WE, Baker RJ (1991) Estimation of heritability and prediction of selection response in plant populations. Crit Rev Plant Sci 10(3):235–322. 10.1080/07352689109382313

[CR60] Park RF, Goyeau H, Felsenstein FG, Bartos P, Zeller FJ (2001) Regional phenotypic diversity of *Puccinia triticina* and wheat host resistance in western Europe, 1995. Euphytica 122:113–127

[CR61] Pathan AK, Park RF (2006) Evaluation of seedling and adult plant resistance to leaf rust in European wheat cultivars. Euphytica 149:327–342

[CR62] Perrier X, Jacquemoud-Collet JP (2006) DARwin software: dissimilarity analysis and representation for Windows. CIRAD, Montpellier, France. Available online: http://darwin.cirad.fr accessed 26 April 2019

[CR63] Poland J, Endelman J, Dawson J, Rutkoski J, Wu S, Manes Y, Dreisigacker S, Crossa J, Sánchez-Villeda H, Sorrells M, Jannink J-L (2012) Genomic selection in wheat breeding using genotyping-by-sequencing. Plant Genome 5:103–113

[CR64] Ponce-Molina LJ, Huerta-Espino J, Singh RP, Basnet BR, Alvarado G, Randhawa MS, Lan CX, Aguilar-Rincón VH, Lobato-Ortiz R, García-Zavala JJ (2018) Characterization of adult plant resistance to leaf rust and stripe rust in Indian wheat cultivar ‘New Pusa 876.’ Crop Sci 58:630–638 10.1094/PDIS-11-16-1545-RE 30673516

[CR65] Pritchard JK, Stephens M, Donnelly P (2000) Inference of population structure using multilocus genotype data. Genetics 155:945–959 10835412 10.1093/genetics/155.2.945 PMC1461096

[CR66] Qiao L, Gao X, Jia Z, Liu X, Wang H, Kong Y, Qin P, Yang B (2024) Identification of adult resistant genes to stripe rust in wheat from southwestern China based on GWAS and WGCNA analysis. Plant Cell Rep 43:67 38341832 10.1007/s00299-024-03148-4

[CR67] R Core Team (2024) R: A language and environment for statistical computing R Foundation for Statistical Computing Vienna

[CR68] Ragagnin VA, De Souza TLPO, Sanglard DA, Arruda KMA, Costa MR, Alzate-Marin AL, Carneiro JEdS, Moreira MA, De Barros EG (2009) Development and agronomic performance of common bean lines simultaneously resistant to anthracnose, angular leaf spot and rust. Plant Breed 128:156–163

[CR69] Rampitsch C, Huang M, DjurishabCignaovic S, Wang X, Fernando U (2019) Temporal quantitative changes in the resistant and susceptible wheat leaf apoplastic proteome during infection by wheat leaf rust (Puccinia triticina). Front Plant Sci 10:1291 31708941 10.3389/fpls.2019.01291 PMC6819374

[CR70] Reif JC, Zhang P, Dreisigacker S, Warburton ML, van Ginkel M, Hoisington D, Bohn M, Melchinger AE (2005) Wheat genetic diversity trends during domestication and breeding. Theor Appl Genet 110(5):859–864. 10.1007/s00122-004-1881-8 15690175

[CR71] Rollar S, Geyer M, Hartl L, Mohler V, Ordon F, Serfling A (2021) Quantitative trait loci mapping of adult plant and seedling resistance to stripe rust (*Puccinia striiformis* Westend) in a multiparent advanced generation intercross wheat population. Front Plant Sci 12:684671 35003147 10.3389/fpls.2021.684671 PMC8733622

[CR72] Schulthess AW, Kale SM, Zhao Y, Gogna A, Rembe M, Philipp N, Liu F, Beukert U, Serfling A, Himmelbach A, Oppermann M, Weise S, Boeven PHG, Schacht J, Longin CFH, Kollers S, Pfeiffer N, Korzun V, Fiebig A, Schüler D, Lange M, Scholz U, Stein N, Mascher M, Reif JC (2022) Large-scale genotyping and phenotyping of a worldwide winter wheat genebank for its use in pre-breeding. Sci Data 9:784. 10.1038/s41597-022-01891-5 36572688 PMC9792552

[CR73] Schulthess AW, Kale SM, Liu F, Zhao Y, Gogna A, Jiang Y, et al. (2021) Genomics-informed parent selection uncovers the breeding value of wheat genetic resources. bioRxiv.2021.12.15.472759. Preprint at 10.1101/2021.12.15.472759

[CR74] Septiningsih EM, Pamplona AM, Sanchez DL, Maghirang-Rodriguez R, Neeraja CN, Vergara GV, Heuer S, Ismail AM, Mackill DJ (2009) Development of submergence tolerant rice cultivars: the Sub1 gene and beyond. Ann Bot 103:151–160 18974101 10.1093/aob/mcn206 PMC2707316

[CR75] Serfling A, Krämer I, Lind V, Schliephake E, Ordon F (2011) Diagnostic value of molecular markers for Lr genes and characterization of leaf rust resistance of German winter wheat cultivars with regard to the stability of vertical resistance. Eur J Plant Pathol 130:559–575

[CR76] Sharma R, Wang M, Chen X, Lakkakula IP, Amand PS, Bernardo A, Bai G, Bowden RL, Carver BF, Boehm JrJD, Aoun M (2025) Genome-wide association mapping for the identification of stripe rust resistance loci in US hard winter wheat. Theor Appl Genet 138:67 40063245 10.1007/s00122-025-04858-3 PMC11893644

[CR77] Shin JH, Blay S, McNeney B, Graham J (2006) LDheatmap: an R function for graphical display of pairwise linkage disequilibria between single nucleotide polymorphisms. J Stat Softw 16:1–9

[CR78] Singh RP, Huerta-Espino J, Bhavani S et al (2011) The emergence of Ug99 races of the stem rust fungus is a threat to world wheat production. Annu Rev Phytopathol 49:465–481. 10.1146/annurev-phyto-072910-095423 21568701

[CR79] Song S, Zhang N, Fan X, Wang G (2025) bHLH transcription factors in cereal crops: diverse functions in regulating growth, development and stress responses. Int J Mol Sci 26:9915 41155209 10.3390/ijms26209915 PMC12563628

[CR80] Tong J, Zhao C, Liu D, Jambuthenne DT, Sun M, Dinglasan E, Periyannan SK, Hickey LT, Hayes BJ (2024) Genome-wide atlas of rust resistance loci in wheat. Theor Appl Genet 137:179. 10.1007/s00122-024-04689-8 38980436 PMC11233289

[CR81] Vazquez MD, Zemetra R, Peterson CJ, Chen XM, Heesacker A, Mundt CC (2015) Multi-location wheat stripe rust QTL analysis: genetic background and epistatic interactions. Theor Appl Genet 128:1307–1318. 10.1007/s00122-015-2507-z 25847212

[CR82] Vikas VK, Pradhan AK, Budhlakoti N, Mishra DC, Chandra T, Bhardwaj SC, Kumar S, Sivasamy M, Jayaprakash P, Nisha R et al (2022) Multi-locus genome-wide association studies (ML-GWAS) reveal novel genomic regions associated with seedling and adult plant stage leaf rust resistance in bread wheat (*Triticum aestivum* L). Heredity 128:434–449 35418669 10.1038/s41437-022-00525-1 PMC9177675

[CR83] Vikram P, Sehgal D, Sharma A, Bhavani S, Gupta P, Randhawa M, Pardo N, Basandra D, Srivastava P, Singh S, Sood T (2021) Genome-wide association analysis of Mexican bread wheat landraces for resistance to yellow and stem rust. PLoS ONE 16:e0246015 33513167 10.1371/journal.pone.0246015 PMC7846011

[CR84] Volk GM, Byrne PF, Coyne CJ, Flint-Garcia S, Reeves PA, Richards C (2021) Integrating genomic and phenomic approaches to support plant genetic resources conservation and use. Plants 10:2260 34834625 10.3390/plants10112260 PMC8619436

[CR85] Walkowiak S, Gao L, Monat C, Haberer G, Kassa MT, Brinton J, et al. (2020) Multiple wheat genomes reveal global variation in modern breeding. Nature 588(7837): 277–283 10.1038/s41586-020-2961-x PMC7759465 33239791

[CR86] Wang L, Xiang L, Hong J, Xie Z, Li B (2019) Genome-wide analysis of bHLH transcription factor family reveals their involvement in biotic and abiotic stress responses in wheat (*Triticum aestivum* L). 3 Biotech 9:236 31139551 10.1007/s13205-019-1742-4 PMC6536565

[CR87] Wang J, Wang J, Li J, Shang H, Chen X, Hu X (2021) The RLK protein TaCRK10 activates wheat high-temperature seedling-plant resistance to stripe rust through interacting with TaH2A 1. Plant J 108:1241–1255 34583419 10.1111/tpj.15513

[CR88] Warnes G, Gorjanc G, Leisch F, Man M (2013) Genetics: Population Genetics R Package Version 1.3–8–1. Available online: http://CRAN.R-project.org/package=genetics. Accessed 22 Sep 2024

[CR89] Wu J, Ma S, Niu J et al (2025) Genomics-driven discovery of superior alleles and genes for yellow rust resistance in wheat. Nat Genet 57:2017–2027. 10.1038/s41588-025-02259-2 40696174

[CR90] Yao F, Guan F, Duan L, Long L, Tang H, Jiang Y, Li H, Jiang Q, Wang J, Qi P, Kang H (2021) Genome-wide association analysis of stable stripe rust resistance loci in a Chinese wheat landrace panel using the 660K SNP array. Front Plant Sci 12:783830 35003168 10.3389/fpls.2021.783830 PMC8728361

[CR91] Ye B, Singh RP, Yuan C, Liu D, Randhawa MS, Huerta-Espino J, Bhavani S, Lagudah ES, Lan C (2022) Three co-located resistance genes confer resistance to leaf rust and stripe rust in wheat variety Borlaug 100. Crop J 10:490–497. 10.1016/j.cj.2021.07.004

[CR92] Zhang P, Yan X, Gebrewahid TW, Zhou Y, Yang E, Xia X, He Z, Li Z, Liu D (2021) Genome-wide association mapping of leaf rust and stripe rust resistance in wheat accessions using the 90K SNP array. Theor Appl Genet 134:1233–1251 33492413 10.1007/s00122-021-03769-3

[CR93] Zhang H, Fechete LI, Himmelbach A, Poehlein A, Lohwasser U, Börner A, Maalouf F, Kumar S, Khazaei H, Stein N, Jayakodi M (2024) Optimization of genotyping-by-sequencing (GBS) for germplasm fingerprinting and trait mapping in faba bean. Legume Sci 6:e254

[CR99] Zhou X, Li X, Han D, Yang Q, Kang Z, Ren J (2022) Genome-wide QTL mapping for stripe rust resistance in the elite wheat cultivar Pindong 34. Front Plant Sci 13:930932 10.3389/fpls.2022.932762 PMC9296828 35873978

[CR94] Zhu T, Wang L, Rimbert H, Rodriguez JC, Deal KR, De Oliveira R, Choulet F, Keeble-Gagnère G, Tibbits J, Rogers J, Eversole K, Appels R, Gu YQ, Mascher M, Dvorak J, Luo M-C (2021) Optical maps refine the bread wheat Triticum aestivum cv Chinese Spring genome assembly. Plant J 107:303–314. 10.1111/tpj.15289 33893684 PMC8360199

[CR95] Zhu Z, Cao Q, Han D, Wu J, Wu L, Tong J, Xu X, Yan J, Zhang Y, Xu K, Wang F, Dong Y, Gao C, He Z, Xia X, Hao Y (2023) Molecular characterization and validation of adult-plant stripe rust resistance gene Yr86 in Chinese wheat cultivar Zhongmai 895. Theor Appl Genet 136:142. 10.1007/s00122-023-04374-2 37247049

